# Menstrual physiology: implications for endometrial pathology and beyond

**DOI:** 10.1093/humupd/dmv038

**Published:** 2015-08-07

**Authors:** Jacqueline A. Maybin, Hilary O.D. Critchley

**Affiliations:** MRC Centre for Reproductive Health, University of Edinburgh, The Queen's Medical Research Institute, 47 Little France Crescent, Edinburgh EH16 4TJ, UK

**Keywords:** endometrium, inflammation, angiogenesis, progesterone, hypoxia

## Abstract

**BACKGROUND:**

Each month the endometrium becomes inflamed, and the luminal portion is shed during menstruation. The subsequent repair is remarkable, allowing implantation to occur if fertilization takes place. Aberrations in menstrual physiology can lead to common gynaecological conditions, such as heavy or prolonged bleeding. Increased knowledge of the processes involved in menstrual physiology may also have translational benefits at other tissue sites.

**METHODS:**

Pubmed and Cochrane databases were searched for all original and review articles published in English until April 2015. Search terms included ‘endometrium’, ‘menstruation’, ‘endometrial repair’, ‘endometrial regeneration’ ‘angiogenesis’, ‘inflammation’ and ‘heavy menstrual bleeding’ or ‘menorrhagia’.

**RESULTS:**

Menstruation occurs naturally in very few species. Human menstruation is thought to occur as a consequence of preimplantation decidualization, conferring embryo selectivity and the ability to adapt to optimize function. We highlight how current and future study of endometrial inflammation, vascular changes and repair/regeneration will allow us to identify new therapeutic targets for common gynaecological disorders. In addition, we describe how increased knowledge of this endometrial physiology will have many translational applications at other tissue sites. We highlight the clinical applications of what we know, the key questions that remain and the scientific and medical possibilities for the future.

**CONCLUSIONS:**

The study of menstruation, in both normal and abnormal scenarios, is essential for the production of novel, acceptable medical treatments for common gynaecological complaints. Furthermore, collaboration and communication with specialists in other fields could significantly advance the therapeutic potential of this dynamic tissue.

## Introduction

The phenomenon of human menstruation has been shrouded in mystery throughout history. Many questions regarding menstrual physiology remain unanswered, not least ‘why does it happen?’ Historically, menstruation has been regarded negatively. Historia Naturalis states ‘Wine sours if they pass, vines wither, grass dies, and buds are blasted. Should a menstruating woman sit under a tree, the fruit will fall. A looking glass will discolour at her glance, and a knife turn blunt’ (Pliny, AD 77–79). Aristotle viewed menstruation as an outward sign of female inferiority, a view that persisted into the nineteenth century and beyond. A leading British psychiatrist in 1874 wrote ‘with one week of the month more or less sick and unfit for hard work she is intellectually handicapped’. A pioneering nineteenth century Scottish gynaecologist claimed, ‘young girls should not play music or read serious books because it makes much mischief with their menstrual cycle’. Hence menstruation was regarded as incapacitating and, in turn, intellect dangerous for menstrual physiology.

These negative connotations of menstruation are inextricably linked to the lower social position of women in society. Currently, global differences in women's rights and status have a dramatic impact on reproductive health and consequently their morbidity and mortality. As women receive high quality education, begin working outside the home, gain the right to vote and have easy access to emergency healthcare and birth control, the ‘taboo’ of menstruation weakens. Therefore, some see the attitude of a society to menstruation as a barometer for civilization and equality. When in the USA in the 1960s, it was suggested that women lacked the ability to hold positions of responsibility and power due to their menstrual cycle, eminent US endocrinologist Estelle Ramage counteracted, ‘In man, the shedding of blood is always associated with injury, disease, or death. Only the female half of humanity is seen to have the magical ability to bleed profusely and still rise phoenix-like each month from the gore’. Despite this positive outlook, historical negative connotations of menstruation still have a significant impact in current society, including the perceptions and expectations of women and their healthcare providers.

However, as women undertake positions of responsibility in the workplace and home, abnormal menstruation can cause significant socio-economic problems. Abnormal menstrual bleeding affects 20–30% of premenopausal women (RCOG, 2011), and more than 800 000 women seek treatment annually in the UK (NICE, 2007). A US study demonstrated financial losses of >$2000 per patient each year due to work absence and home management costs (Frick *et al.*, 2009). Although time has proven that physiological menstruation is not a barrier to female success; family and career responsibilities may become impossible if heavy or painful bleeding occurs. Due to advances in family planning, women in developed countries now can expect greater than 400 episodes of menstruation in their lifetime. This is in stark contrast to our ancestors and women in very underdeveloped countries, who have ∼40 menstrual bleeds due to multiple pregnancy and long spells of lactational amenorrhoea (Short, 1976). In this way, menstrual abnormalities are a relatively modern disorder.

**Figure 1:**
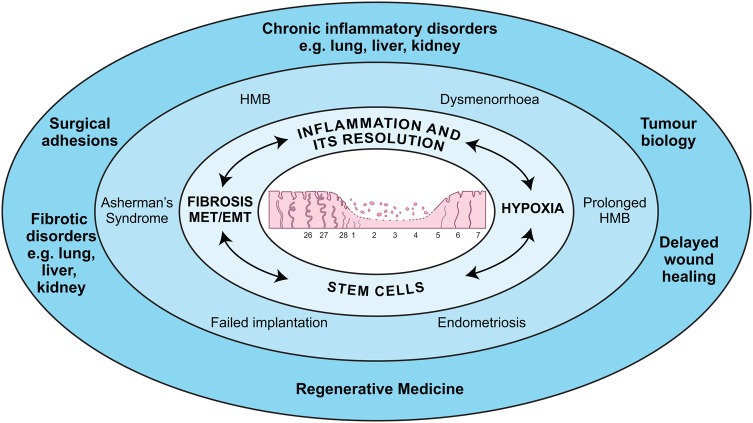
The relevance of menstrual physiology. The perimenstrual endometrium (centre) is exposed to inflammation and hypoxia. Stem cells and EMT are involved at menses to enable scar-free repair (light blue). Aberrations in these processes can lead to gynaecological disorders (mid-blue). Study of endometrial physiology may help delineate the pathogenesis of a number of disorders in other tissue sites (dark blue).

As societies' view of menstruation changes for the better, the views of individual women suffering from common menstrual problems remain understandably negative. This review article aims to provide scientific evidence of both facets of menstrual physiology. First, how normal menstruation could contribute to scientific and clinical breakthroughs in all areas of health and disease and conversely, how aberrations in menstrual physiology can result in significant reproductive disorders with a severe impact on quality of life (Fig. 1). As we detail the physiology of menstruation, we aim to highlight the clinical applications of what we know, the key questions that remain and the scientific and medical possibilities for the future.

## Methods

Pubmed and Cochrane databases were searched for all original and review articles published in English until April 2015. Search terms included ‘endometrium’, ‘menstruation’, ‘endometrial repair’, ‘endometrial regeneration’ ‘angiogenesis’, ‘decidualization’, ‘inflammation’, ‘heavy menstrual bleeding (HMB)’ and ‘menorrhagia’. We reviewed the manuscripts and included them as appropriate.

## Results

### To bleed or not to bleed?

Human females are one of the few species that menstruate, alongside old world primates, elephant shrews and fruit bats. The ovarian steroid hormones regulate endometrial function and human menstruation. After human ovulation, the corpus luteum secretes high levels of progesterone to maintain endometrial receptivity should fertilization occur. In the absence of pregnancy the corpus luteum regresses, causing a sharp decline in circulating progesterone levels. This triggers a local inflammatory response in the endometrium involving infiltration of leukocytes, cytokine release, oedema and activation of matrix metalloproteinases (Jabbour *et al.*, 2006). The result is tissue breakdown and shedding of the upper two-thirds of the endometrium (the functional layer) during the menstrual phase of the cycle (see ‘What causes menstruation’ section). However, in non-menstruating species tissue breakdown and bleeding do not occur in response to progesterone withdrawal. Instead of shedding, considerable remodelling and reabsorption of the endometrium takes place.

Many theories for why women menstruate have been proposed, including defence against pathogens contained in sperm or energy efficiency of shedding versus endometrial maintenance. However, these theories do not account for differences between menstruating and non-menstruating mammals or the evolutionary basis of menstruation (Finn, 1996). Current evidence favours the spontaneous decidualization hypothesis. During the secretory phase (post ovulation until menstruation) of the menstrual cycle progesterone acts upon an estrogen primed endometrium. This causes decidualization; converting the elongated endometrial stromal cells into more spherical decidual cells and increasing their production of prolactin, insulin-like growth factor binding protein-1 (IGBP-1) and glycogen (Brosens *et al.*, 1999; Dunn *et al.*, 2003). Decidualization is initiated by cAMP and occurs in the perivascular stromal cells before spreading in an outward ‘wave’ across the stromal compartment. In women, and indeed all of the menstruating species, decidualization occurs ‘spontaneously’ prior to implantation. In contrast, the endometrium of non-menstruating mammals only undergoes decidualization when there is contact between the embryo and endometrium, i.e. at the time of implantation (Finn, 1998).

There is a strong correlation between the degree of trophoblast invasion during placental development and the extent of decidualization (Finn, 1996). Of note, the human endometrium undergoes the most extensive decidualization process and demonstrates the greatest degree of trophoblast invasion of all species (Ramsey *et al.*, 1976). This extensive and spontaneous decidualization reaction is thought to confer maternal immunotolerance to the partially allogenic embryo, allowing controlled placental invasion (Emera *et al.*, 2012). In addition, spontaneous decidualization may provide a maternal screen for genetically abnormal embryos. Many human pre-implantation embryos contain genetic aneuploidies and chromosomal imbalances, similar to those found in cancer cells. It seems prudent that the maternal environment should provide some selection over the embryos that will invade the endometrium. This hypothesis is supported by findings in women with recurrent miscarriage, where a higher proportion of poor quality embryos implant into a suboptimally decidualized endometrium (Salker *et al.*, 2012). Therefore, menstruation is obligatory in the absence of pregnancy, as spontaneous decidualization of the endometrium has taken place. This may be viewed as an inevitable consequence of reproductive quality control but an additional benefit has also been proposed (Blanks and Brosens, 2013). Repeated shedding of the endometrium necessitates complete repair and regeneration of the denuded surface. Therefore, events that would only otherwise occur after parturition are repeated monthly. This may bestow upon the human endometrium an extraordinary ability to adapt to optimize function and would explain why most women suffering from recurrent miscarriage eventually achieve a successful pregnancy (Blanks and Brosens, 2013). Hence, there may be an evolutionary benefit to menstruation that explains its occurrence, and persistence, in women. So what can we learn from this physiological process that has been so carefully preserved in women and what are the consequences when aberrations occur?

### Menstruation: a model of self-limiting inflammation?

The menstrual endometrium displays the classic hallmarks of inflammation, including tissue oedema and influx of immune cells. This inflammatory process that occurs in the endometrium at menstruation is entirely physiological and tightly regulated to prevent loss of function (Critchley *et al.*, 2001). Outwith the reproductive tract, this physiological inflammation does not occur. However, the ovary and endometrium display repeated inflammation throughout a woman's reproductive lifespan at ovulation and menstruation, respectively (Rae and Hillier, 2005). Delineation of the factors involved and their regulation may lead to therapeutic benefits for gynaecological conditions such as heavy menstrual bleeding (HMB) and may be applicable to a host of inflammatory disorders at other tissue sites.

#### What causes menstruation?

##### Progesterone withdrawal

It is widely accepted that the sharp decline in circulating progesterone levels due to corpus luteum demise is the trigger for menstruation in women. Human studies using progesterone antagonists during the secretory phase have mimicked the events of menstruation, providing proof that progesterone withdrawal is important in menstrual physiology. Administration of mifepristone during the mid-secretory phase has increased our knowledge of local endometrial events during human menstruation, revealing an increase in endometrial inflammatory mediators, such as cyclo-oxygenase (COX-2), nuclear factor (NF)κB and interleukin (IL)-8 (also known as CXCL8) (Critchley *et al.*, 1999a, 2003). Studies in the rhesus macaque have confirmed the importance of progesterone withdrawal in the induction of menstruation (McClellan *et al.*, 1984; Nayak *et al.*, 2000). Menstruation was artificially induced in macaques by surgical removal of ovaries followed by 14 days of estrogen priming prior to insertion of a progesterone capsule to mimic the secretory phase. Consistent with findings in women, removal of the progesterone implant resulted in menstruation, even when estradiol exposure was maintained. This finding emphasizes the dominance of progesterone withdrawal over estradiol withdrawal for menses induction. In contrast, when attempting to induce simulated menstruation in the scientifically versatile murine model, progesterone withdrawal was insufficient for induction of bleeding (Finn and Pope, 1984; Brasted *et al.*, 2003; Menning *et al.*, 2012; Rudolph *et al.*, 2012; Cousins *et al.*, 2014). This problem was surmounted by an injection of arachis oil into the uterine lumen when progesterone levels are high. This ‘induced injury’ resulted in pre-implantation decidualization of the murine endometrium, analogous to naturally occurring mid-secretory events in the macaque and human. Hence, the murine model of simulated menstruation reiterates the importance of decidualization prior to progesterone withdrawal in menstrual physiology.

Further support for the key role of the decidualized endometrial stromal cell in menstrual induction is derived from human *in vivo* and *in vitro* studies. Of note, the progesterone receptor (PR) has at least two isoforms, PRA and PRB, which act as transcriptional regulators of progesterone responsive genes (Graham *et al.*, 1995; Graham and Clarke, 1997). Although the basal endometrial layer shows persistent PR expression throughout the menstrual cycle, PR has differing temporal and locational expression in the functional layer (Lessey *et al.*, 1988; Snijders *et al.*, 1992). PR is widely present during the proliferative phase, but there is a significant decline in glandular epithelial cells of the functional layer during the secretory phase. In contrast, PR persists in the stromal compartment of the functional layer throughout the secretory phase, particularly in the perivascular region. Immunohistochemical analysis of human tissue revealed that PRA is the predominant isoform during the secretory phase, with PRB declining in both stromal and glandular cells in the latter half of the cycle (Wang *et al.*, 1998; Brosens *et al.*, 1999; Mote *et al.*, 2001). Hence, endometrial stromal cells remain responsive to progesterone throughout the secretory phase. Gene microarray-based studies have been reviewed in Dey *et al.* (2004) and showed that analysis of mid-secretory uterine tissue, *ex vivo* progesterone/PR antagonist-treated endometrium, treated *in vitro* decidualized stromal cells and uterine tissue from PR-deficient mice have identified a panel of progesterone responsive genes that may be important for implantation. Hence the mid-secretory phase decidualized stromal cells retain PR expression and confer maximal progesterone responsiveness, priming the endometrium to respond to progesterone withdrawal.

In 2001, Kelly *et al.* published the hypothesis that local endometrial events following progesterone withdrawal occur in two phases (Kelly *et al.*, 2001). The initial phase involves an influx of cytokines and prostaglandins (PG) to the endometrium that is dependent on an efficient response of the perivascular decidualized stromal cells to decreasing levels of the anti-inflammatory hormone progesterone (Catalano *et al.*, 2007; Evans and Salamonsen, 2014). The second phase occurs as a consequence of increased cytokine production and results in an influx of leukocytes to the endometrial environment, activation and release of matrixmetalloproteinases (MMPs) and destruction of the extracellular matrix (ECM). This lytic phase is thought to be independent of PR actions. This hypothesis was supported by an elegant study in ovariectomized macaques, where progesterone implants were removed as normal at the end of the simulated cycle but replaced at staggered time-points from 12 to 72 h after initial withdrawal (Slayden and Brenner, 2006). Replacement up to 24 h after withdrawal prevented menstruation and prevented increases in endometrial MMP1, 2 and 3. Replacement after 36 h had no effect on menstruation and partially blocked MMP production, with significantly less endometrial MMP2 expression. More recently, these findings have been replicated in the murine model of simulated menstruation (Wang *et al.*, 2013). These studies demonstrate a temporal progesterone deprived threshold, over which menstruation becomes inevitable.

##### Endometrial inflammation and leukocyte traffic

Although progesterone withdrawal has an undeniable role in the initiation of menstruation and MMPs are widely accepted as the mediators of endometrial breakdown (Marbaix *et al.*, 1996), the intermediate mechanisms of menstruation remain under investigation. Progesterone withdrawal regulates phase one of menstruation, by up-regulating local cytokine presence (Hannan *et al.*, 2004; Jones *et al.*, 2004). However, phase two occurs despite progesterone replacement after the critical threshold suggesting subsequent, independent regulation. Further evidence for these downstream regulatory factors comes from observational studies of MMP expression in human endometrial tissue. MMPs have the ability to degrade all components of the ECM and are up-regulated at the time of menstruation as a result of progesterone withdrawal (Marbaix *et al.*, 1996; Vassilev *et al.*, 2005). However, MMP expression in the perimenstrual phase is limited to the functional endometrial layer despite the global hormonal changes and persistent PR expression in the basal layer, suggesting a more local tissue site-specific regulation.

Gene microarray analysis of endometrial tissue biopsies collected from women during the mid-secretory phase when compared with those taken following progesterone withdrawal has identified potential gene candidates involved in the regulation of menstruation. These studies revealed an increase in CXCL8 and cyclo-oxygenase (COX)-2 following progesterone withdrawal (Critchley *et al.*, 1999a; Catalano *et al.*, 2007). COX is the rate-limiting enzyme in the synthesis of PG and is present in two isoforms. COX-1 is widely expressed in many tissues, whereas COX-2 is highly inducible. PGE_2_ and F_2α_ have important reproductive functions (Critchley *et al.*, 2006). Loss of EP2, a PGE_2_ receptor, resulted in impaired ovulation and reduced litter size (Kennedy *et al.*, 1999; Tilley *et al.*, 1999). Gene ablation of the FP receptor, the receptor for PGF_2α_, resulted in loss of parturition (Sugimoto *et al.*, 1997). Both PGE2 and PGF2α concentrations are increased significantly in the human during the window of implantation in natural cycles and also in patients undergoing *in vitro* fertilization (IVF) and ovum donation. Interestingly, this profile is abrogated when the endometrium is refractory (Vilella *et al.*, 2013).

*In vitro* studies of decidualized human stromal cells revealed that steroid hormone withdrawal increased a host of inflammatory mediators, including IL-6, CCL11, GM-CSF, CCL2, IL1-RA, CXCL10 and CXCL8, and this response was mediated by NF-κB (Evans and Salamonsen, 2014). NF-κB increases the transcription of a wide variety genes, including cytokines (IL-1, IL-6), chemokines (CXCL8, chemokine ligand 2/CCL-2) and adhesion molecules (intercellular adhesion molecule 1/ICAM, vascular cell adhesion molecule 1/VCAM) (Kayisli *et al.*, 2004). Human endometrial biopsies have also been shown to express components of the NF-κB pathway, with evidence for activation of NF-κB during the perimenstrual phase (King *et al.*, 2001). These findings have been replicated in the mouse menstrual-like model (Xu *et al.*, 2013).

A recent study in the mouse model of simulated menstruation links NF-κB and COX-2 in the menstrual process. Inhibition of the COX enzymes or NF-κB at the time of progesterone withdrawal significantly decreased the amount of bleeding and endometrial breakdown in this murine model (Xu *et al.*, 2013). Furthermore, there was a significant decrease in leukocyte influx after both interventions. Chromatin immunoprecipitation analysis revealed that NFkB binds to the COX-2 promoter, providing a mechanism of NFkB-mediated COX-2 up-regulation and subsequent inflammatory cell influx at menstruation. Progesterone is known to have inhibitory effects on NF-κB activity, mediated by increasing IκB production or by competing with NF-κB for recognition sites on relevant genes (Kelly *et al.*, 2001). In this way, the steroid hormones modulate the local endometrial inflammatory environment by suppressing NF-κB activity until menstruation is required.

Following progesterone withdrawal, there is a dramatic rise in the endometrial leukocyte population (Bonatz *et al.*, 1992; Salamonsen and Lathbury, 2000). Neutrophil numbers are negligible throughout most of the cycle but increase perimenstrually to comprise 6–15% of the total cell number (Salamonsen and Lathbury, 2000). As key mediators of the inflammatory response, neutrophils respond to inflammation by migrating rapidly to the site of injury to contain and clear any noxious stimuli. Circulating neutrophils have a lifespan of a few hours, but neutrophils residing in inflamed tissue can survive for days. This is due to decreased neutrophil apoptosis induced by pro-inflammatory mediators and hypoxia (Ward *et al.*, 1999; Cross *et al.*, 2006). The importance of this neutrophil influx at menstruation was shown in the mouse model, where neutrophil depletion using the antibody RB6-8C5 affected endometrial breakdown and markedly delayed endometrial repair (Kaitu'u-Lino *et al.*, 2007a). Neutrophils contain high levels of MMPs and have the ability to activate resident MMPs to initiate endometrial breakdown (Gaide Chevronnay *et al.*, 2011). In contrast, chronic inflammation is characterized by a persistent neutrophil response due to decreased apoptosis (Serhan and Savill, 2005). This prolonged neutrophil response results in tissue damage and loss of function. Therefore, tight regulation of neutrophil influx and apoptosis is required for normal menstruation. B cell lymphoma 2 (bcl-2) is an apoptosis regulator protein that is expressed in the human endometrium (Otsuki *et al.*, 1994). Examination of human endometrial tissue revealed the presence of bcl-2 during the proliferative and early secretory phases with decreased levels in the late secretory and menstrual phases. These decreased levels correlated with the appearance of apoptotic cells in the perimenstrual phase. This cyclic pattern suggests that ovarian hormones regulate bcl-2 levels in the endometrium (Critchley *et al.*, 1999b). In this way, progesterone withdrawal may increase bcl-2 to limit the lifespan of endometrial neutrophils at menstruation, preventing a chronic inflammatory response.

Macrophages also increase in number throughout the secretory phase to reach maximal numbers perimenstrually, during the luteo-follicular transition (Bonatz *et al.*, 1992; Critchley *et al.*, 1999a; Thiruchelvam *et al.*, 2013). The regulation of the endometrial macrophage remains under investigation. Lacking PR, these cells may be recruited from the circulation due to increased endometrial chemoattractant production and/or may proliferate *in situ* (Guo *et al.*, 2011; Davies *et al.*, 2013b). These cells produce cytokines and proteases and are involved in tissue remodelling and debris removal. The classic M1 (pro-inflammatory) and M2 (anti-inflammatory) phenotypes represent simplified extremes of macrophage function. These complex cells have the ability to adapt and respond to the tissue environment in which they reside (Gordon and Martinez, 2010; Davies *et al.*, 2013a). The phenotype of endometrial macrophages during the perimenstrual phase is yet to be fully delineated, but considering their known functions they are likely to have a significant impact in the endometrium at menstruation (Thiruchelvam *et al.*, 2013). Furthermore, delineation of macrophage phenotype in this physiological model of tissue ‘injury’ and ‘repair’ may provide novel insights to pathological conditions, such as chronic inflammation or cancer, where resident macrophages are involved in aberrant function (Laoui *et al.*, 2014). A direct comparison of the macrophage profile throughout the physiological inflammatory response of menstruation with the macrophage response in areas of chronic inflammation may lead to novel therapeutic targets to improve tissue function.

**Figure 2:**
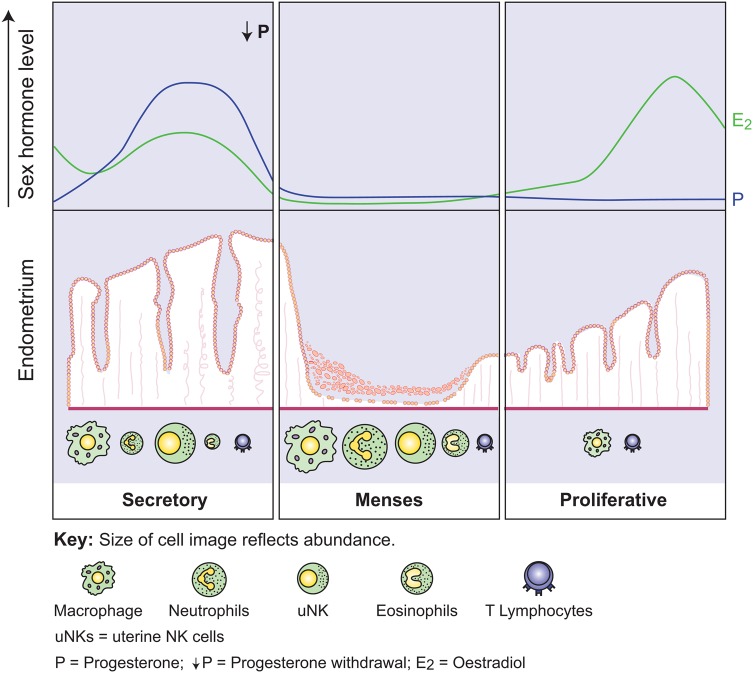
Leukocyte trafficking in the perimenstrual human endometrium (derived from data published and reviews by Bonatz *et al.*, 1992; Salamonsen and Lathbury, 2000; Moffett-King, 2002; Thiruchelvam *et al.*, 2013). Top panel: Sex steroid profiles in the luteo-follicular transition (perimenstrual ‘window’). Bottom panel: Overview of leukocyte traffic in the endometrium with transition from secretory phase through menses/endometrial repair to the proliferative phase of next cycle. Size of cell image reflects abundance.

Taken together, the studies described above support the hypothesis that the decidualized stromal cell compartment can increase cytokine and chemokine production to attract leukocytes, or encourage their proliferation in the functional endometrial layer, during the perimenstrual phase. For summary of perimenstrual leukocyte traffic, see Fig. 2. In turn, endometrial leukocytes produce MMPs and have the potential to stimulate MMP production from adjacent cells (Jabbour *et al.*, 2006) making them attractive candidates for the regulation of local endometrial MMP expression. In this way the decidualized stromal cells of the functional layer help determine their own fate, limiting the inflammatory reaction and tissue breakdown to the upper luminal portion of the endometrium. This compartmentalization of inflammation, with sparing of the basal layer, may be critical for efficient repair of the endometrium after shedding (menstruation). There is evidence that the amount of endometrium that is shed during menstruation varies between individuals, but it remains undetermined if the depth of shedding is associated with gynaecological pathologies (Ludwig and Spornitz, 1991; Fraser *et al.*, 2001).

#### What limits endometrial inflammation?

An excessive or prolonged inflammatory response at menstruation will lead to excessive tissue damage and may result in HMB (NICE, 2007). Studies examining endometrial tissue from women with objective measurement of their menstrual blood loss (MBL) have identified a significantly increased inflammatory response in women with HMB. Increased levels of the pro-inflammatory cytokine tumour necrosis factor α were identified in the menstrual effluent of women with HMB (MBL > 80 ml) compared with women with normal MBL (Malik *et al.*, 2006). Endometrial COX-2 mRNA expression was also significantly elevated in women with HMB (Smith *et al.*, 2007). In addition, increased levels of total PGs have been found in the endometrium of women with HMB (Smith *et al.*, 1981a, b). Furthermore, increased signalling of PGE_2_ through its EP2 and EP4 receptors has been suggested due to elevated production and decreased hydrolysis of cyclic AMP (Smith *et al.*, 2007). In support of these findings, PG synthesis inhibitors are a popular treatment for HMB. Mefenamic acid is a non-steroidal anti-inflammatory compound which significantly decreases MBL (Cameron *et al.*, 1990). However, although women treated with mefenamic acid have a significant decrease in their menstrual loss, 52% maintained a blood loss greater than 80 ml after 2 months of treatment, highlighting the need for more effective medical therapies for this condition (Cameron *et al.*, 1990).

##### Glucocorticoids

The inflammatory response of physiological menstruation appears to be self-limiting. The pro-inflammatory cytokine IL-1 has been shown to increase the expression of 11β hydroxysteroid dehydrogenase-1 (11βHSD-1) (Rae *et al.*, 2004; Rae and Hillier, 2005). This enzyme converts cortisone (compound E) to the anti-inflammatory steroid cortisol (compound F). Glucocorticoids alter the inflammatory response by limiting cytokine production, increasing macrophage phagocytosis, increasing transcription of anti-inflammatory genes and repressing pro-inflammatory transcription factors (Zhang *et al.*, 2009).

Endometrial 11βHSD-1 mRNA levels are significantly increased at menstruation, consistent with a role in endometrial breakdown and repair (McDonald *et al.*, 2006). In addition, the glucocorticoid receptor is present throughout the cycle in the stromal compartment, including endometrial leukocytes and endothelial cells (Bamberger *et al.*, 2001; Henderson *et al.*, 2003). In this way, local generation of glucocorticoids by inflammatory mediators may prevent an excessive inflammatory response in the menstrual endometrium. Studies of endometrium from women with HMB further highlight the importance of glucocorticoids in endometrial physiology. Secretory endometrium from women with a blood loss greater than 80 ml was found to have significantly elevated levels of 11βHSD-2 when compared with endometrium from women with normal loss (Rae *et al.*, 2009). 11βHSD-2 converts cortisol back to cortisone and may explain the excessive local inflammation of the endometrium in women with HMB at menses. Decreased cortisol levels and loss of its anti-inflammatory effects may prolong menses, contributing to heavy blood loss. We are currently exploring whether ‘rescue’ of putative luteal phase endometrial glucocorticoid deficiency could reduce menstrual bleeding (Warner *et al.*, 2015).

##### Control of the MMP response at menses

MMPs have the ability to degrade all components of the ECM and have been shown to have an integral role in endometrial breakdown at menses (Marbaix *et al.*, 1996). Lack of control of MMP action at menstruation will lead to excessive tissue damage and may lead to abnormal bleeding. The control of MMP action occurs at a number of levels to prevent an abnormal response during menses and allow for tissue regeneration and remodelling at other phases of the cycle. A full review of these processes is beyond the scope of this review, and the reader is referred to Gaide Chevronnay *et al.* (2011) for a comprehensive overview. It is well established that progesterone inhibits MMP transcription to suppress their expression during the secretory phase of the cycle (Schatz *et al.*, 1994; Salamonsen *et al.*, 1997; Vassilev *et al.*, 2005). The withdrawal of progesterone and the up-regulation of MMP levels during menstruation have been discussed above. Following endometrial breakdown, MMP activity can be inhibited by tissue inhibitors of metalloproteinases (TIMPS) or by the protease inhibitor α_2_-macroglobulin. These factors are expressed in the endometrium throughout the menstrual cycle (Sayegh *et al.*, 1995; Zhang and Salamonsen, 1997) suggesting that they are overwhelmed by an increase in MMP production at menstruation and that the ratio of MMPs to TIMPs may dictate the ability of MMPs to breakdown tissue. Additionally, active MMPs undergo endocytic clearance by low-density lipoprotein receptor-related protein-1 (LRP-1) during the proliferative and secretory phase of the cycle, initiating lysosomal degradation. At menstruation, the LRP-1 protein is not present due to tissue shedding (Selvais *et al.*, 2009), enhancing MMP activity. This multifactorial regulation limits the MMP response to menstruation, ensuring tissue damage is not prolonged.

### The endometrium: a model of vascular function

#### Menstruation as a physiological ischaemia-reperfusion injury

The first observations of endometrial architecture at menstruation were from intraocular endometrial transplants in the rhesus macaque (Markee, 1940). Direct observation of the explants following progesterone withdrawal revealed shrinkage of endometrial thickness, followed by vasoconstriction of spiral arterioles and focal bleeding. The vasoconstriction observed was transient but intense, consistent with an ischaemia-reperfusion injury. However, the presence and role of hypoxia in the endometrium remain controversial.

Ischaemia has not been detected in the human endometrium during menstruation to date. Laser Doppler fluximetry measures the number of red blood cells transiting a monitored volume per unit time. This method failed to detect ischaemia during menstruation (Gannon *et al.*, 1997), but the limited spatial resolution of fluximetry may not detect focal or prolonged ischaemia-reperfusion episodes. There is some indirect evidence that hypoxia occurs at menstruation in human endometrial tissue. Markers of tissue hypoxia (CAIX and hypoxia inducible factor (HIF)-1α) have been detected immunohistochemically in the human endometrium at menses, with a distinct reduction in staining of both markers after cycle day 5 (Critchley *et al.*, 2006; Punyadeera *et al.*, 2006). In addition, hypoxia has been detected in the menstrual endometrium of the simulated mouse menstruation model (Fan *et al.*, 2008). Pimonidazole is a marker of oxygen partial pressures less than 10 mmHg, and its expression was seen in the uppermost endometrial zones during the simulated menstrual phase. Negligible pimonidazole levels were observed by Day 5 after progesterone withdrawal. In contrast, hypoxia, pimonidazole and HIF-1α were not detected following ovarian hormone withdrawal in a xenograft menses model, where a fragment of human endometrial functional layer was grafted into immunodeficient mice (Coudyzer *et al.*, 2013). These differences may be explained by disturbance of the full thickness endometrial architecture in the immunodeficient xenograft model, where spiral arteriole function and immune cell function will be modified, but definitive proof that hypoxia is present in the human endometrium at menses is still lacking.

Even if hypoxia is present in the endometrium, there remains debate about its function. Primary human endometrial stromal cells cultured in normoxic (21% O_2_) and hypoxic (2% O_2_) conditions for 24 and 48 h revealed that hypoxia decreased the secretion of membrane-type 1 MMP, active MMP-2, proMMP-1 and proMMP-3 (Zhang and Salamonsen, 2002). Similar decreases in MMPs were also observed in the culture supernatants from whole endometrial explants cultured in 0.1% O_2_ for 24 h (Gaide Chevronnay *et al.*, 2010). This suggests that hypoxia is not involved in endometrial breakdown by MMPs at menstruation, but does not exclude a role in repair of the denuded surface and limitation of the MMP response. In the xenograft model described previously, increases in MMP expression were observed in the human endometrial grafts and breakdown occurred within 96 h of ovarian hormone withdrawal. In addition, the xenografted endometrium underwent complete repair despite the absence of hypoxia. This suggests that hypoxia is not essential for endometrial breakdown or repair. However, *in vivo* human menstruation occurs 48–72 h after withdrawal of ovarian hormones (Catalano *et al.*, 2007) and the mouse model of menstruation demonstrates bleeding within 8–12 h of hormone withdrawal (Brasted *et al.*, 2003; Menning *et al.*, 2012; Cousins *et al.*, 2014). Hence 8 h post-progesterone withdrawal in the murine model is approximately equivalent to 48 h in the human. Therefore, it remains possible that, although hypoxia is not necessary for endometrial breakdown and repair, it is desirable for maximal efficiency of these processes. HIF-1 is a transcription factor known to be the master regulator of the cellular response to hypoxia (Iyer *et al.*, 1998). In hypoxic conditions, this factor increases the transcription of a number of genes involved in angiogenesis, mitogenesis and metabolism. Its prolonged activation is observed in the tumour microenvironment, leading to aberrant angiogenesis and metastasis (Mazzone, 2010). However, transient activation appears necessary in physiological situations to instigate repair processes. For example, pharmacological activation of HIF-1 provided protection against development of colitis in a murine model (Cummins *et al.*, 2008). The role of HIF-1 in menstruation, if any, remains to be determined.

#### Vasoconstriction

Regardless of the presence or absence of hypoxia in the menstrual endometrium, vasoconstriction of spiral arterioles is desirable at this time to limit blood flow. Poiseuille's equation states that the radius of a vessel is the major determinant of resistance to flow, meaning that a small increase in vessel radius will dramatically increase the amount of blood flowing through it (Maybin *et al.*, 2011a). Therefore, decreased constriction of endometrial vessels at the time of menstruation will contribute significantly to increased menstrual blood loss. PGF_2α_ and endothelin-1 (ET-1) are two endometrial factors with known vasoconstrictive properties (Baird *et al.*, 1996; Marsh *et al.*, 1997). In contrast, PGE_2_ is a known vasorelaxant. Women with heavy MBL have been shown to have a significantly decreased PGF_2α_/PGE_2_ ratio (Smith *et al.*, 1981b) and decreased FP receptor expression (Smith *et al.*, 2007). Excessive PGE_2_ production at the expense of PGF_2α_ may result in less constriction of the spiral arterioles prior to menstruation. In addition, women with HMB have decreased endometrial expression of the potent vasoconstrictor ET-1 and increased expression of its metabolising enzyme, neural endopeptidase (Marsh *et al.*, 1997). Increased metabolism of endothelin could explain its decreased endometrial expression and cause dilation of endometrial vessels at menstruation. Furthermore, altered spiral arteriole maturation may also contribute to inefficient spiral arteriole vasoconstriction at menstruation. Vessel wall circumference and focal discontinuities were noted to be larger in the endometrium of women with HMB than normal controls (Mints *et al.*, 2007). Women with heavy bleeding had significantly reduced vascular smooth muscle cell proliferation in spiral arterioles during the mid-late secretory phase when compared with normal controls (Abberton *et al.*, 1999b). In addition, smooth muscle myosin heavy chain, a contractile protein used as a marker of vascular smooth muscle cell maturation, was significantly decreased in spiral arterioles of women with HMB (Abberton *et al.*, 1999a). The endothelial cell lining in endometrial tissue from women with HMB was found to have increased gaps, possibly due to increased expression of angiopoietin-2 during the secretory phase (Mints *et al.*, 2010). This suggests that vessels in these women are pre-programmed during the proceeding cycle to be more fragile at menstruation. Taken together, the decreased levels of vasoconstrictive factors and immature vessels present in women with HMB will significantly increase MBL.

#### The endometrial coagulation system

**Figure 3:**
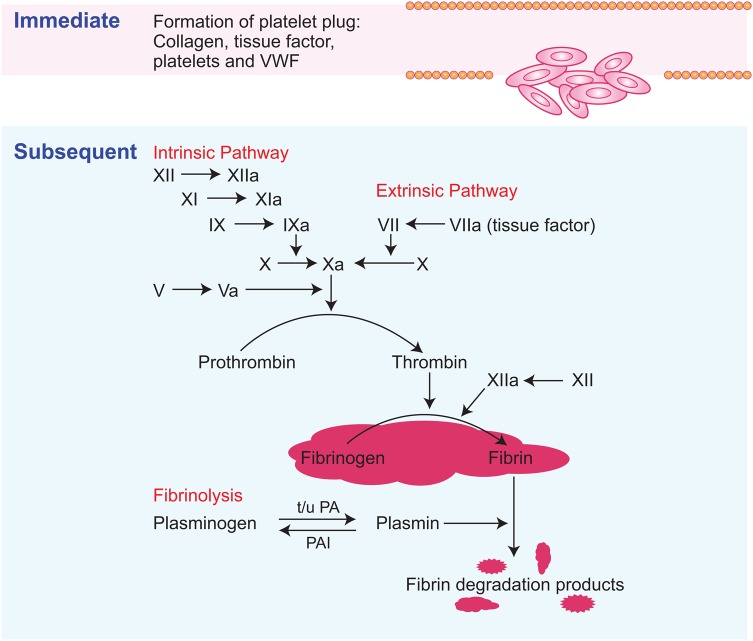
Endometrial coagulation pathways. Immediate: A platelet plug forms rapidly, relying on interactions with tissue factor, vWF and collagen. Subsequent: intrinsic and/or extrinsic activation of coagulation pathways result in formation of a fibrin clot to ensure haemostasis. Fibrinolysis drives the degradation of the fibrin clot. t-PA and u-PA convert plasminogen to plasmin, which breaks down the fibrin clot. PAI converts plasmin back to plasminogen.

Cessation of menstruation relies on an intact endometrial coagulation system to achieve haemostasis (Fig. 3). Endometrial endothelial injury initiates immediate activation and aggregation of platelets to form a plug. This takes place by two mechanisms (i) platelet glycoprotein interaction with von Willebrand factor (vWF) or (ii) tissue factor generation of thrombin (Davies and Kadir, 2012). The resulting platelet plug forms a barrier to prevent further blood loss. The subsequent stage of haemostasis involves the formation of fibrin via the coagulation cascade. The coagulation cascade is activated by two pathways; extrinsic and intrinsic. Each culminates in the conversion of factor X to Xa, which catalyses the conversion of pro-thrombin to thrombin, ultimately leading to the formation of a more stable fibrin clot to seal previously bleeding vessels. Disorders that interfere with systemic haemostasis have an impact on MBL. Von Willebrand disease is the most common of these disorders, with a prevalence of 13% in women with a complaint of HMB (Shankar *et al.*, 2004).

Fibrinolysis involves conversion of plasminogen to active plasmin, promoting the degradation of fibrin deposits. Tissue plasminogen activator (t-PA) and urokinase plasminogen activator (u-PA) drive the production of plasmin. In contrast, plasminogen activator inhibitor (PAI) inhibits fibrinolytic activity. The human endometrium contains t-PA and u-PA, as well as PAI and the uPA receptor (Gleeson *et al.*, 1993; Nordengren *et al.*, 2004). There is evidence that an overactive fibrinolytic system interferes with haemostasis and contributes to HMB. Women with HMB had raised levels of t-PA activity on the second day of bleeding compared with those with normal loss (Gleeson *et al.*, 1993). The efficacy of tranexamic acid as a treatment for HMB provides further evidence for over activation of the fibrinolytic system in the endometrium of these women. This antifibrinolytic reduces t-PA and PAI levels in women with HMB and results in a 58% reduction in blood loss (Gleeson *et al.*, 1994).

#### Angiogenesis

Vascular modification and new blood vessel growth are essential components of endometrial physiology. At menstruation, rapid repair of injured blood vessels must occur to stop bleeding, and this is usually completed by Day 5 of the cycle. This process occurs despite lack of ovarian hormone support, as observed in women following surgical ovariectomy who stop bleeding despite the lack of ovarian hormonal support. In addition, the murine model of menstruation displayed complete repair of the endometrium in the absence of estradiol (Kaitu'u-Lino *et al.*, 2007b), suggesting vascular repair at menses (in this animal model) is not reliant on estrogen. The regulation of vascular repair at this stage is still to be fully delineated. Vascular endothelial growth factor (VEGF), a key mediator of vascular function, is increased in women at menses, and there is mounting evidence from human and murine studies that endometrial VEGF is regulated by hypoxia (Charnock-Jones *et al.*, 1993; Sharkey *et al.*, 2000; Fan *et al.*, 2008; Maybin *et al.*, 2011b).

During the proliferative phase, there is rapid growth of the functional layer of the endometrium, necessitating angiogenesis to maintain perfusion of new tissue (Girling and Rogers, 2005). This physiological angiogenic response is unusual in the human adult and provides an accessible human model for comparison to pathological situations such as the tumour microenvironment. Therefore, defining the control and mechanisms of this normal angiogenesis may identify new approaches to the control of tumour growth. Despite the significant changes in endometrial architecture across the cycle, it has been repeatedly demonstrated that levels of endothelial cell proliferation within the human endometrium do not show any consistent pattern across the menstrual cycle (Girling and Rogers, 2005). Interpretation of these results is challenging, as samples taken from women at the same stage of the menstrual cycle are extremely variable. This may be due to differences in hormone levels at the time of sampling or a variation in the region from which the biopsy was obtained. To overcome this, Nayak and Brenner utilized the macaque simulated menses model (Nayak and Brenner, 2002). Using Ki67 or bromodeoxyuridine (BrdU) to identify proliferating endothelial cells, the authors demonstrated a 6-fold increase in proliferation during the mid-proliferative stage (Days 8–10 after progesterone withdrawal). This peak was absent in the hormone-deprived animals, indicating endothelial cell proliferation at this stage in the cycle is estradiol dependent, unlike the vascular response at menses. No significant changes in proliferation were observed at other stages of this artificial cycle. This finding concurs with stereological analysis of human endometrial endothelial cell staining, where vascular length density was greatest during the mid-late proliferative phase (Gambino *et al.*, 2002). By examining vessel length and branch points, the authors concluded that vessel elongation is the major mechanism by which endometrial angiogenesis occurs in mid-proliferative phase.

In the secretory phase, coiling and maturation of the spiral arterioles and growth of the subepithelial capillary plexus must take place. The consequence of impaired vascular maturation during this phase has been discussed above in regard to decreased vasoconstriction and its relation to HMB. It is likely that the uterine natural killer (uNK) cells play an important role in spiral arteriole maturation.

Uterine NK cells are CD56^bright^, CD16-, CD3- and are phenotypically different from peripheral blood NK cells (Koopman *et al.*, 2003). uNK cells increase in number during the mid-luteal phase and are located close to the endometrial glands and spiral arteries (Dosiou and Giudice, 2005), supporting a role in vascular remodelling. Evidence derived from early pregnancy studies supports a role for uNK cells in the early stages of spiral artery remodelling, where failure of this process is considered to contribute to pregnancy pathology (Moffett-King, 2002; Robson *et al.*, 2012). The lack of spiral arteriole modification observed in mice deficient in uNK cells further supports this hypothesis (Greenwood *et al.*, 2000; Ashkar *et al.*, 2003).

Additional evidence for the impact of uNK cells on endometrial vasculature comes from studies of the action of selective progesterone receptor modulators (SPRMs) in the human endometrium. Analysis of endometrium from women administered the SPRM asoprisnil revealed a suppressed IL-15 pathway, which regulates uNK development and function. There was a marked reduction in uNK cells, an abnormal appearance of endometrial vasculature with increased α-SMA staining surrounding the spiral arterioles. Women taking this SPRM had significantly decreased menstrual bleeding, linking the PR, uNK cells, vascular structure and menstrual function (Wilkens *et al.*, 2013). The inability to identify the PR on uNK cells (Henderson *et al.*, 2003) suggests an indirect mechanism of hormonal regulation, via paracrine mediators such as chemokines (Salamonsen and Lathbury, 2000; Hannan and Salamonsen, 2007).

The importance of vascular normalization has recently become apparent in the field of cancer biology. Blockade of VEGF to prevent angiogenesis in the tumour microenvironment was logically introduced as a treatment for cancer (Carmeliet, 2005). Although initial results were encouraging, the mean survival of patients treated with these inhibitors disappointingly remained unchanged (Carmeliet and Jain, 2011). Recent research has highlighted the benefits of vessel normalization, rather than inhibition of angiogenesis, as a mechanism to reduce metastasis and hopefully increase survival (Mazzone *et al.*, 2009; Carmeliet and Jain, 2011). Therefore, delineation of normal vascular processes and their regulation within the human endometrium, including physiological angiogenesis and vessel maturation, could have widespread clinical application.

### The perimenstrual endometrium: a model of scarless tissue repair

After shedding its luminal portion, the endometrium must efficiently repair to ensure implantation can take place if fertilization occurs in the subsequent cycle. The processes involved in endometrial repair appear to be analogous to classic wound healing and include inflammation, its resolution, angiogenesis, tissue formation and tissue remodelling. The first three processes have been discussed above, and this section will concentrate on the latter two, with discussion of the former where necessary. The cross disciplinary benefits of studying this scar-free repair system are obvious, but incisive data on the factors involved and their regulation remain elusive and concerted efforts are necessary to maximize the translational benefits.

#### The regulation of endometrial repair and regeneration

Scanning electron microscopy of human menstrual endometrial samples revealed a ragged and torn surface with gland openings and a lack of epithelial covering (Ludwig and Spornitz, 1991). Subsequent regrowth of the epithelium occurred before stromal expansion, with epithelial cells growing from the necks of the glands to meet migrating cells from other glands, forming a new luminal surface. This began on menstrual Day 2, and full coverage of the lumen was achieved by Day 6. A more recent study found that the functional endometrial layer displays simultaneous shedding and repair in a piecemeal fashion during menstruation (Garry *et al.*, 2009). Both of these studies suggest that initial re-epithelialization of the endometrium occurs during active bleeding in the absence of ovarian hormones, consistent with findings in the murine menstruation-like model (Kaitu'u-Lino *et al.*, 2007b) and in women post-oophorectomy.

Tissue recombination studies in the mouse model suggest that uterine epithelialization is required before the stromal compartment can respond to ovarian steroids (Bigsby, 2002). Stromal cell mitoses first appear on Days 5–6 of the human menstrual cycle, when estradiol levels are rising and the epithelial layer has completely healed (Ferenczy *et al.*, 1979). Unlike the initial repair phase, this endometrial regeneration is dependent on ovarian hormone support. VEGF, a potent mitogenic and angiogenic factor, was found to have three peaks of expression in the ovariectomized macaque model of menstruation (Nayak and Brenner, 2002). These increases in VEGF mRNA occurred in the surface epithelium during the early proliferative phase, in the stroma during the mid-proliferative phase and in the glands during the late secretory phase. Comparison of hormone-deprived and estrogen-exposed animals revealed that estrogen is not essential for the early proliferative phase peak but is necessary for VEGF mRNA up-regulation in mid-proliferative stromal cells. These findings support an estrogen-independent initial repair phase and estrogen-dependent regeneration of the endometrium.

Hence alongside initiation of menstruation, progesterone withdrawal is also likely to trigger endometrial repair. Support for this hypothesis is found in gene microarray analysis of differentially expressed transcripts from human endometrial explants cultured *in vitro* in the presence of ovarian hormones or in the absence of hormonal support. This hormone deprivation model revealed ‘wound healing and inflammation’ as a top scoring biological process (Gaide Chevronnay *et al.*, 2010). The importance of VEGF for luminal re-epithelialization and angiogenesis at menstruation was demonstrated using VEGF Trap in the macaque and murine models (Fan *et al.*, 2008). Progesterone withdrawal has been shown to increase the expression of VEGF in the macaque model (Nayak and Brenner, 2002), murine model (Fan *et al.*, 2008) and in human endometrial explants (Maybin *et al.*, 2011b). Hypoxia and PGs have been associated with these increases in VEGF expression (Fan *et al.*, 2008; Maybin *et al.*, 2011b) and may represent downstream mediators of progesterone withdrawal.

The importance of the vascular niche in tissue regeneration is further supported by studies of stromal-derived growth factor (SDF-1) and its receptors CXCL4 and CXCL7. SDF-1 is present throughout the menstrual cycle and CXCR4 expression peaks in the early proliferative phase and is present in epithelial cells and endothelial cells (Laird *et al.*, 2011). SDF has been shown to increase endometrial epithelial cell proliferation *in vitro* (Tsutsumi *et al.*, 2011). A recent study combining an inducible endothelial-cell-specific mouse gene deletion strategy and complementary models of acute and chronic liver injury revealed that differential recruitment of pro-fibrotic CXCR4 or pro-regenerative CXCR7 signalling determines if the liver regenerates or becomes fibrotic after injury. Hence, autocrine signals from the endothelium may influence the rate and nature of the repair process. The role and regulation of CXCR4 and CXCR7 in the normal menstrual endometrium, where scarring is absent, and in the rare syndrome of endometrial scarring, Asherman's, remains to be determined.

The cellular and molecular mechanisms governing epithelial cell proliferation and migration after menstruation have not been fully elucidated. At least three hypothesized mechanisms exist, including (i) proliferation of luminal epithelial cells from the base of the epithelial glands, (ii) mesenchymal to epithelial transition of residual stromal cells and (iii) regeneration of the luminal epithelium from endometrial stem cells.

#### Mesenchymal-to-epithelial transition

Previously, the ‘free-edge’ effect was thought to be responsible for endometrial re-epithelialization, where the absence of neighbouring cells at the wound margin acts as a growth signal (Heimark and Schwartz, 1985). However, scanning electron microscopy of menstrual endometrium revealed that epithelial cells appeared to arise from underlying stromal cells in denuded portions, rather than solely from the necks of epithelial glands (Ludwig and Spornitz, 1991; Garry *et al.*, 2009). This suggests that endometrial stromal cells are reprogrammed at menstruation to lose their mesenchymal cell characteristics and gain epithelial cell traits, a process known as mesenchymal-to-epithelial transition (MET). Evidence for MET during endometrial repair comes from the murine model of simulated menses, where co-expression of the epithelial marker pancytokeratin and the stromal cell marker vimentin occurred in endometrial cells after 24 h of hormone withdrawal (Patterson *et al.*, 2013). Gene microarray analysis of murine uterine tissue from the simulated menses model taken pre- and post-progesterone withdrawal revealed significant changes in genes known to be involved in MET such as cytokeratin, Wnt1, E-cadherin and osteopontin (Cousins *et al.*, 2014). This study also identified actively proliferating cells in the stromal compartment, where there was loss of luminal epithelial coverage and proliferation of adjacent luminal epithelial cells, consistent with simultaneous MET and epithelial cell migration. In this way, the residual basal layer of the endometrium and the adjacent unshed functional layer can contribute to re-epithelialization of the denuded surface. The contribution of the functional endometrial layer to menstrual repair is supported by microarray study of stromal and glandular cells from the basal and functional layer obtained by laser capture microdissection (Gaide Chevronnay *et al.*, 2009). This revealed that in addition to up-regulation of transcripts involved in tissue degeneration, stromal cells from the functional layer also displayed increased levels of genes associated with ECM biosynthesis, indicating an important contribution to repair of adjacent denuded areas.

The reverse process of epithelial-to-mesenchymal transition (EMT) is also important for wound healing, embryogenesis and fibrosis (Gonzalez and Medici, 2014). The loss of adhesion molecules and tight junctions alongside increased expression of mesenchymal cell markers allows migration into tissues. In the embryo, cycles of EMT and MET are necessary for development and highlight the reversibility of these processes (Nieto, 2013). The role of EMT, if any, in the endometrium remains to be determined, but it is likely that a balance of EMT and MET is important for repair processes. Excessive EMT has been implicated in fibrotic diseases of the kidney and lung (Kothari *et al.*, 2014). This may be due to the generation of extreme myofibroblasts that are resistant to apoptosis. Synthesis and remodelling of the ECM by fibroblasts is essential for wound healing. Fibroblasts differentiate into myofibroblasts during the last phases of wound healing and increase their expression of smooth muscle actin (SMA). These myofibroblasts initiate wound contraction and secrete type I collagen. Persistence of myofibroblasts at an injury site results in scar formation (Hantash *et al.*, 2008). Therefore, excessive EMT may induce scaring via aberrant myofibroblast differentiation causing persistence at the injury site. Cytokines, hypoxia, growth factors and components of the ECM have all been implicated in the regulation of EMT (Gonzalez and Medici, 2014). Strict control of these factors in the human endometrium may therefore underpin its exceptional ability to heal without scarring. Interestingly, normal human endometrial stromal cells have significantly less α-SMA expression and contractility when compared with endometriotic stromal cells (Yuge *et al.*, 2007). This suggests endometrial cells have less myofibroblastic differentiation, leading to a reduction in scar formation. In this way, the balance of MET and EMT may influence endometrial repair at menstruation. Aberrations in their control could lead to pathology such as endometriosis, with its associated adhesions and scarring, or delayed endometrial repair and its consequent increased MBL.

#### Stem cells

An alternative, or perhaps complimentary, method of endometrial repair is regeneration of tissue from stem cells or progenitor cells. Evidence of their existence in the endometrium comes from colony-forming units derived from human endometrial samples (Gargett *et al.*, 2009). These cells fulfilled the criteria of self-renewal, high proliferative potential and multilineage differentiation. In addition, the mouse model of simulated menstruation suggests that re-epithelialization of the uterine surface arises from progenitor cells residing in the glandular epithelial cells (Kaitu'u-Lino *et al.*, 2010). Unlike human studies, it is possible to utilize the label retaining technique in the murine model, identifying stem cells due to their relative quiescence and comparatively slower proliferation than more differentiated cells. A pulse of BrdU is followed by a chase period, when slowly cycling cells retain the BrdU label and transient amplifying cells proliferate rapidly and dilute the label. Examination of BrdU and proliferating cell nuclear antigen immunofluorescence in this model revealed that glandular cells proliferated selectively during repair and BrdU labelling remained constant. In contrast, luminal cells showed rapid dilution of BrdU at menstruation. Both epithelial and stromal label retaining cells have been identified in this mouse model (Chan and Gargett, 2006).

For a comprehensive review of the contribution, derivation and application of endometrial stem cells, we refer the reader to a number of papers (Gargett and Masuda, 2010; Cervello *et al.*, 2011, 2013; Deane *et al.*, 2013). Many questions remain, but it is clear that the multipotent potential of cells within the endometrium can have widespread benefits. Endometrial biopsies are obtainable in an outpatient setting, usually without the need for anaesthetic. This is in contrast to the painful bone marrow biopsy used to obtain haematopoietic stem cells. Mesenchymal stem cells obtained from the endometrium are highly proliferative (Gargett *et al.*, 2009) and are therefore attractive for *in vitro* expansion and use in cell-based therapies. Furthermore, multipotent cells have also been derived from menstrual effluent, negating the need for any biopsy (Ulrich *et al.*, 2013). Increased understanding and utilization of these unique endometrial cells will benefit many gynaecological conditions. Endometriosis is caused by implantation and growth of endometrial deposits in other tissue sites and is thought to occur secondary to retrograde menstruation. However, although retrograde menstruation occurs in many women, only ∼10% have evidence of endometrial deposits (Gargett *et al.*, 2014). The prevalence or activity of endometrial stem cells in the endometrial fragments spilling into the abdominal cavity may explain this discrepancy. An insufficiently thick endometrium can contribute to sub-fertility and failed IVF. Endometrial stem cell therapy is a potential treatment to regenerate the endometrium and increase fertility rates in the future (Cervello *et al.*, 2013). In addition, menstrual derived cells have displayed regenerative properties at other tissue sites. They have incorporated into atrophied skeletal muscle fibres in a mouse model of Duchenne muscular dystrophy and have resulted in improved cardiac tissue function in an infarction model (Cui *et al.*, 2007; Toyoda *et al.*, 2007; Hida *et al.*, 2008). Endometrial cells have differentiated into morphologically and functionally glucose-responsive insulin secreting cells, providing a potential therapeutic strategy for diabetes (Santamaria *et al.*, 2011). Therefore, accessible multipotent cells from the endometrium could have widespread and significant future clinical applications.

## Conclusions

Many advances have been made to increase our knowledge of menstrual physiology. However, why women menstruate and what starts, limits and stops menstrual blood loss remain key questions. The endometrium functions as a complex multicellular structure that involves interactions of immune, endocrine and vascular systems. The strict regulation of this tissue to allow cyclical ‘injury’ and ‘repair’ at menstruation results in a remarkable physiological response that allows pregnancy to occur. This accessible tissue, alongside robust animal models, provides a fantastic resource in which to study inflammation, angiogenesis and tissue repair (Fig. 1) to identify new therapeutic targets for gynaecological conditions and generate translational knowledge for application at a host of other tissue sites.

## Authors’ roles

J.A.M wrote the manuscript with supervisory support from H.O.D.C.

## Funding

We acknowledge the following funding from the Medical Research Council (G0000066, G0500047; G0600048; MR/J003611/1) and the Wellcome Trust (083908/Z/07/Z, 100646/Z/12/Z) for support of several studies wherein data derived have been described in this review. Funding to pay the Open Access publication charges for this article was provided by the Wellcome Trust.

## Conflict of interest

J.M.: no conflict of interest. H.O.D.C.: research grant support from Bayer Pharma AG; and consultancy for Bayer Pharma AG, PregLem SA, Gedeon Richter, Vifor Pharma UK Ltd, and AbbVie Inc.
